# The new paradigm of psychiatry precision medicine and its emerging clinical framework

**DOI:** 10.1192/j.eurpsy.2024.1389

**Published:** 2024-08-27

**Authors:** B. Abdelmoula, N. Bouayed Abdelmoula

**Affiliations:** ^1^Genomics of Signalopathies at the service of Precision Medicine - LR23ES07, Medical University of Sfax, Sfax, Tunisia

## Abstract

**Introduction:**

Precision medicine is a promising approach to improving the prevention, prediction and treatment of disease, based on individual characteristics and biomarkers/genetic variants shared by specific subgroups of patients.

**Objectives:**

This study aims to address the new paradigm of precision medicine in psychiatry and to discuss, through the literature, its emerging clinical framework.

**Methods:**

We conducted an exhaustive review of the scientific literature using PubMed database and Google Scholar, with “Precision Medicine in Psychiatry” as keywords.

**Results:**

Our review revealed that while psychiatrists have long practiced a personalized therapeutic approach with, for example, treatment choices guided by individual criteria, the methods for achieving this objectively have until now been largely lacking. This dilemma has begun to be resolved with the implementation of data analysis methods such as machine learning and large-scale genomic analysis studies. The goals of precision psychiatry involved the delineation of genetic risk factors using GWAS, the redefinition of the functional domains involved in mental disorders and pharmacological repositioning. The highly polygenic nature of mental disorders and the failure of GWAS to confirm the role of candidate genes have suggested that a systems genetic approach that considers function at the network level would provide a better approach to the problem of linking heterogeneous genetic risk factors and brain mechanisms. In addition, the growing evidence that certain disorders such as psychotic disorders are syndromes rather than diseases in their own right suggests that many conditions currently recognized as such may have similar underlying patterns of cognitive dysfunction and neurobiological abnormalities that will need to be reclassified.

**Conclusions:**

The application of precision medicine in psychiatry is still in its infancy. Numerous research programs creating large multimodal databases with multiple data on brain imaging, genetics, etc. will soon support the clinical deployment of precision medicine in psychiatry.

**Disclosure of Interest:**

None Declared

